# Deploying Machine and Deep Learning Models for Efficient Data-Augmented Detection of COVID-19 Infections

**DOI:** 10.3390/v12070769

**Published:** 2020-07-16

**Authors:** Ahmed Sedik, Abdullah M Iliyasu, Basma Abd El-Rahiem, Mohammed E. Abdel Samea, Asmaa Abdel-Raheem, Mohamed Hammad, Jialiang Peng, Fathi E. Abd El-Samie, Ahmed A. Abd El-Latif

**Affiliations:** 1 Department of the Robotics and Intelligent Machines, Kafrelsheikh University, Kafrelsheikh 33511, Egypt; ahmedsedik93@gmail.com; 2 Electrical Engineering Department, Prince Sattam Bin Abdulaziz University, Al-Kharj 11942, Saudi Arabia; 3 School of Computing, Tokyo Institute of Technology, Yokohama 226-8502, Japan; 4 School of Computer Science and Technology, Changchun University of Science and Technology, Changchun 130022, China; 5 Department of Mathematics and Computer Science, Faculty of Science, Menoufia University, Shebin El-Koom 32511, Egypt; basma.rahiem@science.menofia.edu.eg; 6 Centre for Excellence in Cybersecurity, Quantum Information Processing, and Artificial Intelligence, Menoufia University, Shebin El-Koom 32511, Egypt; 7 Medical Imaging and Interventional Radiology Departement, National Liver Institute, Menoufia university, Shebin El-Koom 32511, Egypt; mohamed.elsayed1167@liver.menofia.edu.eg; 8 Public Health and Community Medicine Department, Faculty of Medicine Menoufia University, Shebin El-Koom 32511, Egypt; asmaa_dawood@yahoo.com; 9 Information Technology Department, Faculty of Computers and Information, Menoufia University, Shebin El-Koom 32511, Egypt; mohammed.adel@ci.menofia.edu.eg; 10 School of Data Science and Technology, Heilongjiang University, Harbin 150080, China; jialiangpeng@hlju.edu.cn; 11 Department of Electronics and Electrical Communications Engineering, Faculty of Electronic Engineering, Menoufa University, Menouf 32952, Egypt; fathi_sayed@yahoo.com; 12 School of Information Technology and Computer Science, Nile University, Giza 12588, Egypt

**Keywords:** COVID-19, Corona virus, machine learning, deep learning, CNN, LSTM networks, image processing

## Abstract

This generation faces existential threats because of the global assault of the novel Corona virus 2019 (i.e., COVID-19). With more than thirteen million infected and nearly 600000 fatalities in 188 countries/regions, COVID-19 is the worst calamity since the World War II. These misfortunes are traced to various reasons, including late detection of latent or asymptomatic carriers, migration, and inadequate isolation of infected people. This makes detection, containment, and mitigation global priorities to contain exposure via quarantine, lockdowns, work/stay at home, and social distancing that are focused on “flattening the curve”. While medical and healthcare givers are at the frontline in the battle against COVID-19, it is a crusade for all of humanity. Meanwhile, machine and deep learning models have been revolutionary across numerous domains and applications whose potency have been exploited to birth numerous state-of-the-art technologies utilised in disease detection, diagnoses, and treatment. Despite these potentials, machine and, particularly, deep learning models are data sensitive, because their effectiveness depends on availability and reliability of data. The unavailability of such data hinders efforts of engineers and computer scientists to fully contribute to the ongoing assault against COVID-19. Faced with a calamity on one side and absence of reliable data on the other, this study presents two data-augmentation models to enhance learnability of the Convolutional Neural Network (CNN) and the Convolutional Long Short-Term Memory (ConvLSTM)-based deep learning models (DADLMs) and, by doing so, boost the accuracy of COVID-19 detection. Experimental results reveal improvement in terms of accuracy of detection, logarithmic loss, and testing time relative to DLMs devoid of such data augmentation. Furthermore, average increases of 4% to 11% in COVID-19 detection accuracy are reported in favour of the proposed data-augmented deep learning models relative to the machine learning techniques. Therefore, the proposed algorithm is effective in performing a rapid and consistent Corona virus diagnosis that is primarily aimed at assisting clinicians in making accurate identification of the virus.

## 1. Introduction

Corona virus disease 2019 or simply COVID-19 is a potentially severe acute respiratory infection caused by a strain of the Sarbeco virus subgenus SARS-CoV-2 that belongs to the Coronaviridae family [1]. It is the seventh Corona virus known to infect humans [1,2], and despite its similarity to the Severe Acute Respiratory Syndrome (SARS) Corona viruses traced to bats, clinically, it differs from both SARS-CoV-1 and the Middle East Respiratory Syndrome (MERS-CoV) [2]. Alarm regarding this virus is traced to an outbreak of pneumonia of unknown cause in Wuhan City of Hubei Province in China in December 2019. The clinical presentation is a respiratory infection with symptom severity ranging from a mild common cold-like illness to a severe viral pneumonia leading to acute respiratory distress syndrome that is potentially fatal [3]. Transmission of SARS to humans was traced from civet cats (in China in 2002 [4]). The Corona virus strain that causes MERS was traced to camels (in Saudi Arabia in 2012 [5]). Epidemiologically, there are many other known Corona virus strains that circulate among animals without evidence of transmission to humans until now [6]. 

Clinically, as established via genetic sequencing, the new Corona virus is believed to be associated with animals, because most of the initial cases were associated with the marine and animal market in Wuhan, China [6]. This provides subsisting links to the zoonotic origin of the virus. While the potential animal reservoir and intermediary host(s) are unknown at this point, studies suggest the new Corona virus may have originated from a recombination between a bat Corona virus and an origin-unknown Corona virus [7]. The virus is transmitted between humans from an infected person to another through proximity contact without protection [8]. 

More recent data suggests that the virus may be airborne and have a short lifetime [9]. Therefore, two main elements of COVID-19 transmission are respiratory and contiguity. Respiratory droplets are generated when an infected person exhibits respiratory symptoms (for example, sneezing, coughing, etc.), whence a person in close contact is at risk of being exposed to potentially infective respiratory droplets [1]. These droplets may also land on surfaces, where the virus could remain viable; thus, the infected individual and his/her immediate environment can serve as a source of transmission, which is known as contact transmission [10].

Typical symptoms of COVID-19 include fever, cough, shortness of breath and oftentimes the infection develops into pneumonia. It may also cause severe complications for people with weak immunity systems, the elderly, and the people with pre-existing chronic diseases such as cancer, diabetes, and chronic lung disease [11]. Therefore, both the World Health Organisation (WHO) and the Centre for Disease Control (CDC) have established that early detection and containment of this virus are necessary to efficiently curtail the spread of this pandemic [12].

Meanwhile, the potency and utility of Machine Learning (ML) methods has seen an explosion of its use across numerous medical fields, including classification of cardiovascular diseases [13,14], classification of diabetic retinopathy types [15], and classification of corneal patterns [16]. These methods have shown potential in reducing medical errors, early detection or tracking of asymptomatic carriers as well as techniques to enhance disease treatment and provision of effective healthcare to patients. Notwithstanding their utility, ML methods are known to have limitations related to:

- Manual extraction and selection of features.
- Poor performance when dealing with imbalanced datasets.
- Over-fitting.
- Complexity and time consumption.

For their part, DLMs, such as CNNs [17,18,19] and ConvLSTM [20,21,22], have been widely applied in several medical fields as improvements to ML techniques. While ML techniques and DLMs may seem instinctive candidates in our present crusade to annihilate COVID-19, the absence of reliable data to exploit the “learnability” inherent to DLMs makes them palpable choices.

Generative adversarial networks (GANs) have emerged as the preferred technique to address data unavailability across a wide range of learning paradigms. However, many classes of GANs are known to experience instability during the training phase and gradient saturation [23]. Nevertheless, it remains a veritable tool for applications that require data augmentation.

Consequently, in this study, we ameliorate data unavailability via two data augmentation strategies. First, we employ a set of simple image transformations to distend a limited dataset by ten folds to levels effective for learnability by the DLMs. Second, we utilise the potency of GANs for data augmentation. Following both data augmentation steps, we utilise two deep learning models (DLMs) (i.e., Convolutional Neural Network (CNN) and the Convolutional Long Short-Term Memory (ConvLSTM)) in image-based detection of COVID-19.

The rest of this study is structured as follows. Section 2 presents the architecture of our CNN and ConvLSTM algorithms as well as the data augmentation process that is discussed to enhance the performance of the models. The experimental requirements to establish the performance of our models as well as discussions of the outcomes are presented in Section 3.

## 2. Data Augmentation and Efficient Learning by DLMs

Faced with an existential threat, humanity must put its best feet forward with any and every solution to support detection, diagnosis, and treatment of COVID-19. Meanwhile, machine and deep learning techniques have proven potent in numerous medical techniques [13,14,15,16,17,18,19,20,21,22], and epidemiological techniques [24,25]. This study supports engineering and computational science efforts in our collective battle to defeat the COVID-19 scourge. In this section, we present details of two DLMs for detection of the COVID-19 virus from medical images.

Like any ML technique or DLM, given image-based modalities, our proposed models are taught to discriminate between negative and positive tests for COVID-19. However, absence or unreliability of such data impedes the potency of DLMs due to absence of adequate features. This study presents two data augmentation techniques based on simple image transformations and GANs, which has been used in COVID-19 detection based on chest X-ray images [26]. However, in this study, both data augmentation strategies are executed on X-ray and CT images for enhanced COVID-19 detection. 

Consequently, our study presents data-augmented DLMs that despite constrains of lack of and/or limited size of data (or unreliability) are still capable of producing accurate and efficient results. Both data augmentation processes are followed by the use of two DLMs, i.e., CNN and ConvLSTM, for COVID-19 status detection. These two DLMs consist of the phases enumerated in the sequel, which make our data-augmented deep learning models introduced in subsequent subsections.

Data pre-processing phase. This phase enhances the size and quality of the available dataset. Technically, in our case, this entails distending the deficient dataset into a capacious one. This is accomplished via the use of different transformations, such as scaling, rotation and flipping on the available images. In this manner, each image can produce new ones, each with valuable information to support latter stages of our DLM architecture. As a first step, all images are uniformly resized to generate tensors that represent a generalisation of matrices and vectors that can be used for further feature extraction. 

Feature extraction phase. In this phase, various features to support the learning process of the proposed machine and deep learning models are determined and extracted. The large pool of images emanating from the pre-processing phase provides an abundance of learnable features, which feed the classifier. Considering its central role, this phase is critical to the success of the proposed technique. As outlined in the introductory comments of this study, the proposed CNN and the ConvLSTM-based DLMs consist of five convolutional layers and one convolutional layer, respectively, each followed by Max. pooling operations. These architectures support the needed learning to discern the COVID-19 status of the given input image.

Classification phase. The classifier uses feature sequences emanating from the feature extraction to represent the input data. Consequently, there is no positive and negative cases needed for further transformations since the generated feature maps are one-dimensional. As a result, the Global Average Pooling (GAP) is used in both proposed DLMs.

### 2.1. Data Augmentation Process Based on Image Transformations

**Figure 1:**
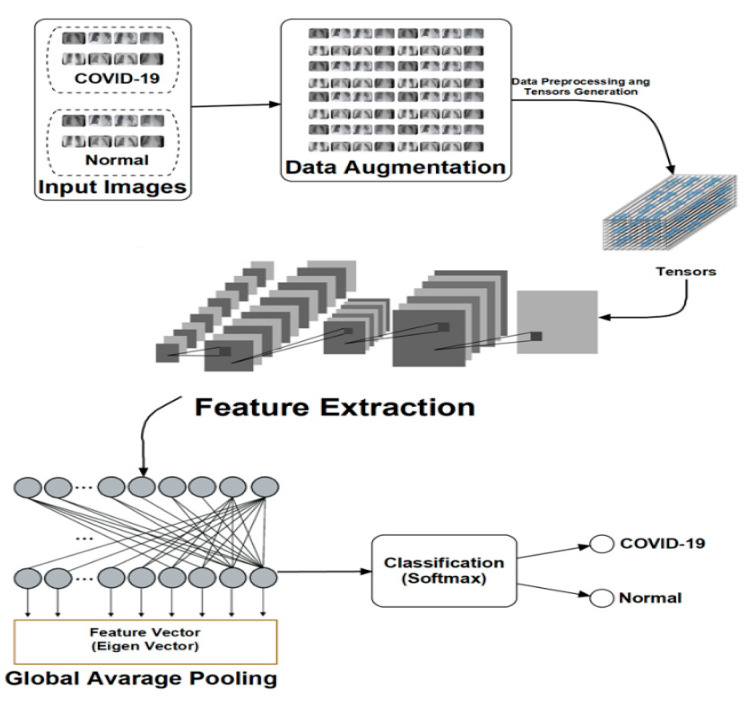
Architecture showing data flow in the proposed image transformation deep learning models (DLMs).

Our first data augmentation strategy involves the use of facile image transformations (scaling, rotation (using different angles) and flipping operations) to proliferate the dataset. In this study, each image is augmented into 10 images via 90-, 80-, and 270-degree rotations, resizing using 0.25, 0.5, 2, 3, 4, and 5 scaling factors and flipping. Consequently, the hitherto limited dataset of 50 images (25 for both COVID-19 negative and positive outcomes) distends into 500 images. Furthermore, each image is converted into a tensor, which is an important step in its subsequent use in both (i.e., CNN and ConvLSTM) DLMs. Figure 1 presents the architecture and data flow for the proposed technique. As seen therein, input images are propagated to provide the needed learning required by the DLMs. The simplicity of this approach as well as the integration of data augmentation into the DLMs motivates the naming (DADLM) for these data-augmented DLMs. 

**Figure 2:**
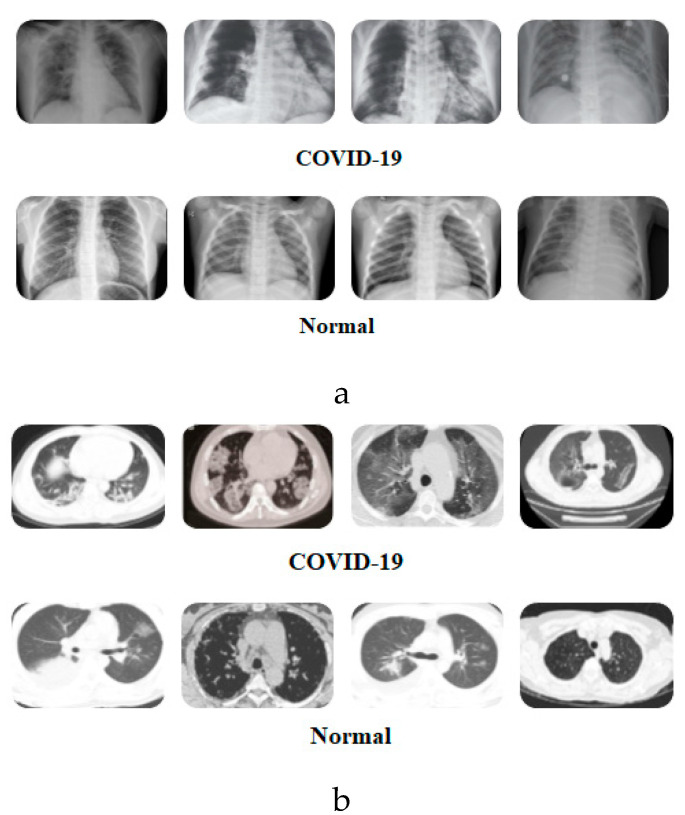
Sample X-ray images [27] and CT images [28] showing COVID-19 positive and negative outcomes. (**a**) Samples of pre-labelled COVID-19 Positive and Normal X-ray images. (**b**) Samples of pre-labelled COVID-19 Positive and Normal CT images.

As mentioned earlier, both of the proposed DADLMs (i.e., CNN DADLM and ConvLSTM DADLM) are also implemented on two sets of data consisting X-ray and CT images pre-labelled as COVID-19 positive and negative. The first dataset comprises 56 X-ray images equally divided and pre-labelled as COVID-19 positive and negative classes that are distended 10 folds to realise 2880 COVID-19 positive (i.e., infected) and the same number of negative (i.e., normal) images. Figure 2 presents samples of images from the two datasets.

### 2.2. Convolutional GAN

Given a training set, the generative adversarial network (GAN) facilitates learning to generate new data with the same statistics as the training set [29]. Since their emergence in 2014, GANs have emerged as the preferred technique to ameliorate data unavailability with applications across wide-ranging learning paradigms be it semi-supervised, supervised or reinforcement [30].

Traditionally, a GAN model consists of two stages: generator and discriminator. The generator network generates feature maps from the input images, while the discriminator reconstructs these feature maps and discriminates between the real and generated images using a classification layer [25]. Since the objective is to generate images rather than to obtain a decision, unlike standard use of GANs, our study limits its use to the data augmentation process by precluding the need for its use in classification. Specifically, our study uses a convolutional GAN (i.e., CGAN), which exacerbates many of the difficulties plaguing GAN training, including instability and gradient saturation [30].

**Figure 3:**
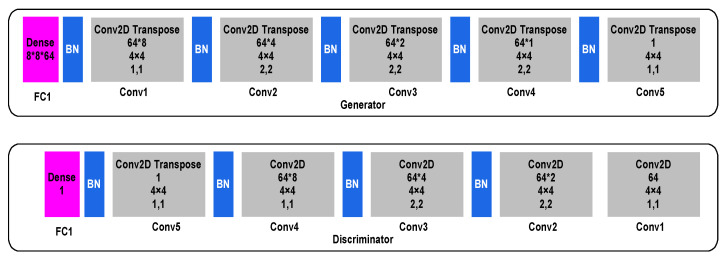
Outline of data flow convolutional generative adversarial network (CGAN) data augmentation.

In CGAN, the generator phase consists of five convolutional transpose layers (Conv2D Transpose)-Conv1, Conv2, Conv3, Conv4 and Conv5, each consisting of 8, 4, 2, 1, and 1 filter, respectively. The input images are first enrolled into a denoising fully connected layer that is primed at a size of 8 × 8 × 64, following which a sequence of Conv2D Transpose layers and batch normalisation (BN) layers are applied to generate a feature map of the input images. At the other end, in the discriminator phase, five convolutional layers (Conv2D), Conv1, Conv2, Conv3, Conv4, and Conv5 each consisting of 64, 2, 4, 8, and 1 filter, respectively, are used to generate a feature map that is enrolled into a sequence of Conv2D and BN layers required to reconstruct the original image. Finally, a denoising fully connected layer is applied on the reconstructed image. Figure 3 outlines the CGAN data augmentation.

Following the CGAN data augmentation, the two DLMs (i.e., CNN and ConvLSTM) are used to facilitate discrimination of the COVID-19 status of an input modality. The integration of CGAN data augmentation into the DLMs motivates the naming CGAN DADLM. Specifically, this study proposes CNN CGAN DADLM and ConvLSTM CGAN DADLM for the CNN and ConvLSTM DLMs, respectively. Technical details of the two DLMs are highlighted in the next subsection.

### 2.3. Technical Specifications of DLMs

#### 2.3.1. ConvLSTM

**Figure 4:**
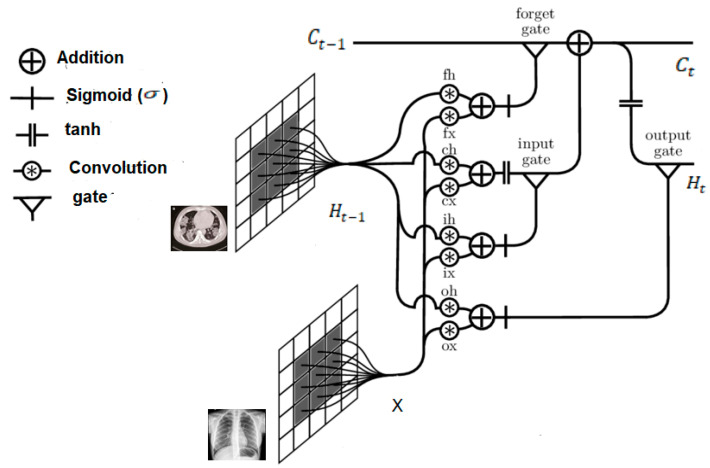
Architecture of the Convolutional Long Short-Term Memory (ConvLSTM).

The Long Short-Term Memory (LSTM) is a special class of the artificial Recurrent Neural Networks (RNNs) and, as with the standard RNN structure, it is proven to be stable and reliable for long-range dependencies and applications in various studies [31]. A drawback of this structure, however, is the redundancy for spatial data. Convoluted LSTM (i.e., ConvLSTM) is used to overcome this problem. Structurally, it replaces the fully connected layers in the LSTM with convolutional layers. If states are viewed as they would in the hidden representations of moving objects, the larger transitional kernel of ConvLSTM should be capable of capturing faster motions, as depicted in Figure 4. To ensure that the states have the same dimensions as their inputs, padding is undertaken prior to applying the convolution operation. All states are initialised to zero before the first input, which corresponds to the so-called “total ignorance” of the future [32,33,34]. The ConvLSTM has the ability to encode the spatio-temporal information in its memory cell. Equations (1) to (5) formalise the mathematical rigours of the ConvLSTM.

(1) $$\;i_{t}=\sigma \left(W_{xi}X_{t}+W_{hi}H_{t-1}+W_{ci}\circ \;C_{t-1}+b_{i}\right)$$

(2) $$f_{t}=\sigma \left(W_{xf}X_{t}+W_{hf}H_{t-1}+W_{cf}\circ \;C_{t-1}+b_{f}\right)$$

(3) $$C_{t}=f_{t}\circ \;C_{t-1}+i_{t}\circ \tanh\left(W_{xc}*X_{t}+W_{hc}H_{t-1}+b_{c}\right)$$

(4) $$\;o_{t}=\sigma \left(W_{xo}X_{t}+W_{ho}H_{t-1}+W_{co}\circ \;C_{t}+b_{o}\right)$$

(5) $$H_{t}=o_{t}\circ \;\tanh\left(C_{t}\right)$$

where $\;i_{t}\;$ is the input gate, $\;C_{t-1}$ is the status at the previous cell, $f_{t}$ is the forget gate, $H_{t}\;$ is the final state of the latest state $C_{t}$, $\;o_{t\;}$ is the output gate, and   ∘  is the Hadamard product.

The above architecture (i.e., Figure 4) elucidates previous assertions regarding use of the larger transitional kernel of the ConvLSTM to capture slight variations. Further, as already mentioned, to ensure that the states have the same dimensions as their inputs, they are padded before applying the convolution operation, and subsequently, all states are initialised as zero. 

#### 2.3.2. The Convolutional Layer

**Figure 5:**
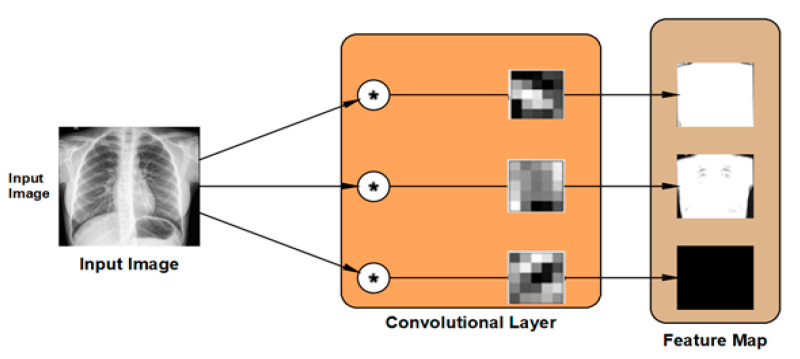
Outline of feature map generation in the convolution layer.

The convolutional layer is the second type of feature extraction tools deployed in the proposed DLMs. The convolutional layer is used to extract the feature map from the input images by applying two-dimensional digital filters. The weights of these digital filters are set randomly in order to transform the input image into its feature map as illustrated in Figure 5. The DLM contains the hierarchical layout of feature maps generated from each layer. Consequently, the new value of a certain pixel can be calculated as:

(6) $$p_{new}=\sum_{i\in s}^{}p_{i}\cdot w_{i}$$

In addition, an activation function is required in order to evaluate the final output of each layer. Our model is executed using the ReLU activation function, whose output can be obtained in the form presented in (7).

(7) $$f\left(x\right)=\underset{}{\max}\left(0,x\right)$$

#### 2.3.3. Pooling Layer

**Figure 6:**
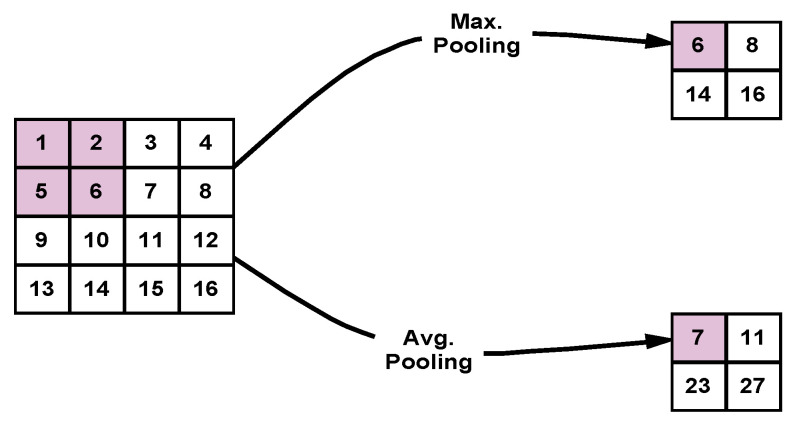
Illustration of Max. and Avg. pooling used in deep learning models (DLMs).

The pooling layer is the third kind of feature extraction tools deployed in the proposed DLMs. Each convolutional layer is followed by a pooling layer that serves to reduce the size of the feature map resulting from the convolutional layer. There are two main types of pooling processes, Max. pooling and Avg. pooling. In Max. pooling, the maximum value of each window is selected, while in Avg. pooling, the mean value of the window is selected. Figure 6 illustrates the Max. and Avg. pooling operations.

### 2.4. Classification Network

The classification network consists of two layers. The first layer is the fully connected layer, which is required to convert the feature map generated by the feature extraction layers into a feature vector that subsequently serves as an input of the classification layer. The second layer is the classification that is carried out by deploying a dense layer with a SoftMax activation function. The output probability of this layer can be obtained as presented in (8).

(8) $$P\left(y=j|x\right)=\frac{e^{x^{T}w_{j}}}{\sum_{k=1}^{K}e^{x^{T}w_{k}}}$$

### 2.5. Network Training 

Like in [29], for network training, we use the Adaptive Moment Estimation (ADAM) optimiser [35], which has a cross-entropy loss function defined as:

(9) V(f(x),t)=−t.ln(f(x))−(1−t)ln(1−f(x))

where the alternative label convention  (t)=(1+y)/2, and t∈{0,1} is used.

The cross-entropy loss function compares each element in the vector containing the standard values after converting it into one-hot encoding and the corresponding element in the vector containing the probability values that come out of the SoftMax function. The closer the two values are, the closer the result will be to zero if there is a match between the labels prediction and standard in the training data. The more the training is conducted in the correct direction, the more the closeness between the expected value and the standard value and, therefore, the less error expected. This process is ubiquitous in modern deep neural networks. 

**Figure 7:**
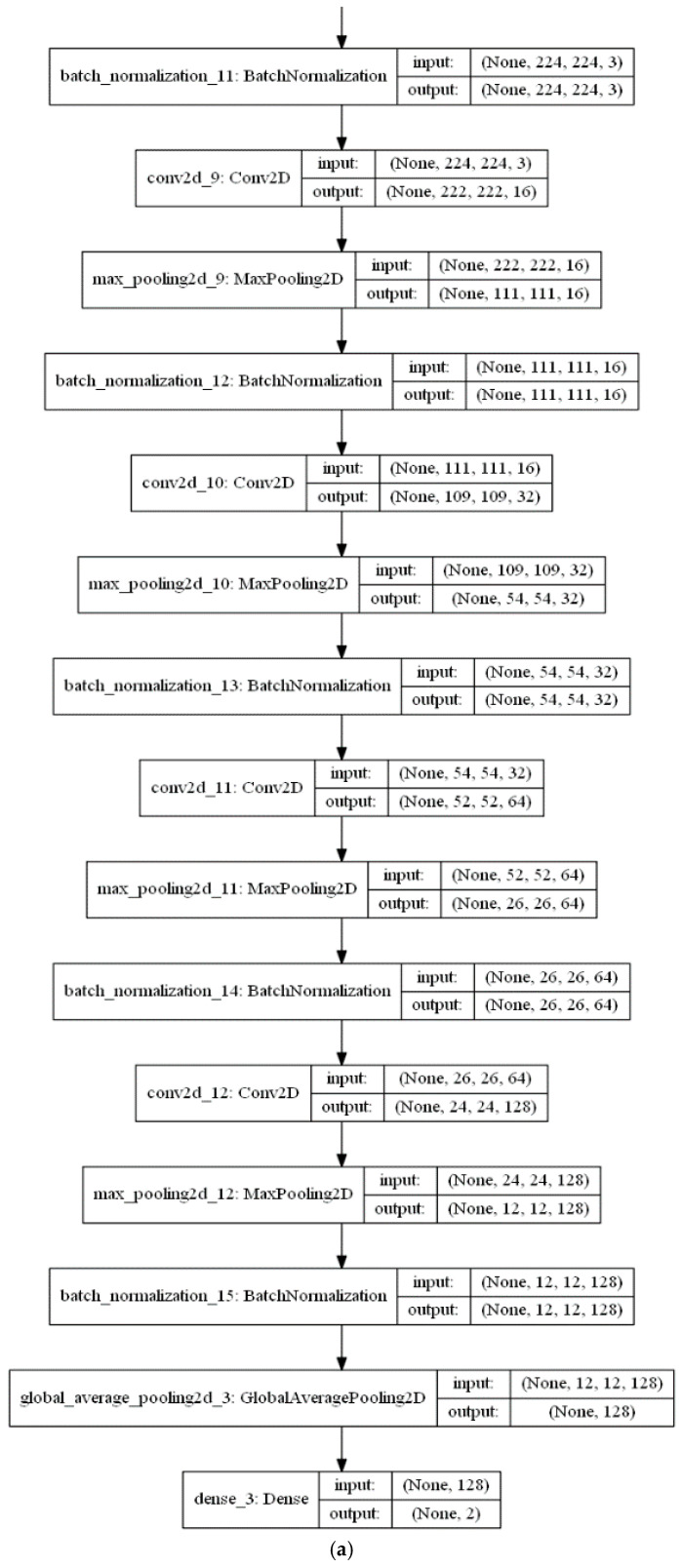

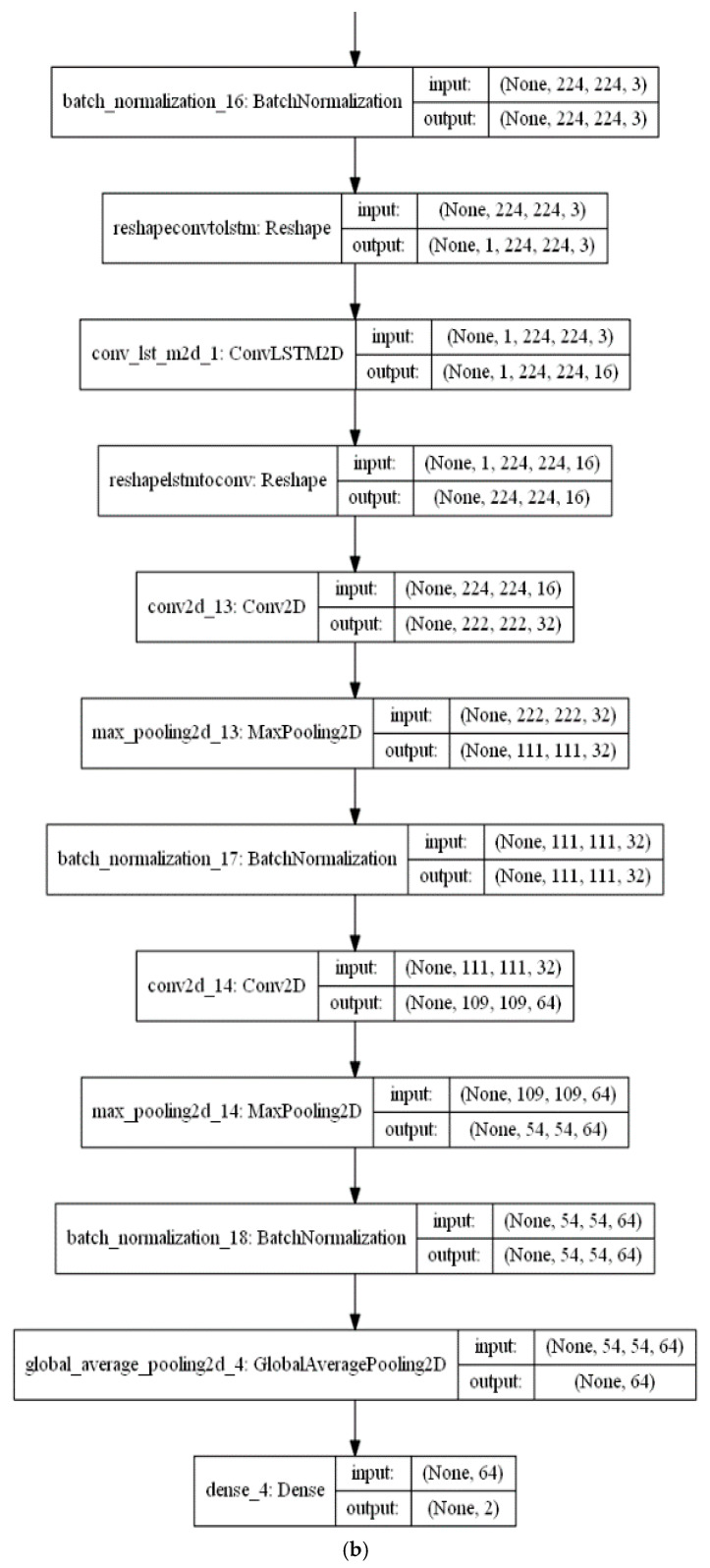
Technical specifications of the proposed deep learning models: (**a**) CNN-based DLM and (**b**) ConvLSTM-based DLM.

The feature extraction stage of the proposed algorithm is performed with a series of a ConvLSTM layer and three Convolutional (CNV) layers, each followed by a Max. pooling layer. Subsequent to these layers, a global average pooling (GAP) acts on the fully connected layer. Finally, a dense layer with size of two classes (i.e., positive (normal) and negative (infected)) is utilised for the classification decision in the classification phase. The CNV layers act as feature extractors, where each CNV layer applies its specific number of filters and produces its feature maps. Beginning with the feature maps produced from the first CNV layer, the subsequent Max. pooling layer produces resized pooled feature map that acts as input to the next CNV layers. The final pooled feature maps of the last Max. pooling layer are rearranged as vectors and inserted into the GAP layer. Figure 7a,b presents a visual representation of technical specifications of the proposed DLMs.

## 3. Experimental Validation

This section presents experiments and performance analysis to validate the efficiency of the proposed techniques for COVID-19 detection. We present the technical specifications of the proposed DLMs, the metrics used to evaluate them as well as the outcomes and discussions about their performance. The simulation experiments are carried out using Python 3.5 programming language. The proposed DLMs are built using Keras deep learning library, while both SVM and *k*-NN are executed using scikit-learn, while the simulation experiments are implemented via GPU interfaces.

### 3.1. Evaluation Metrics

Our study is primarily focused on estimating the efficiency of the two proposed data-augmented DLMs, i.e., using both the simple image transformations and CGAN data augmentation strategies (i.e., DADLM and CGAN DADLM), in discriminating between COVID-19 status (positive and negative cases) of the given images. Therefore, accuracy-based metrics to assess efficiency in terms of accuracy of detection, logarithmic loss, and testing time will be used. These metrics are defined in the remainder of this subsection.

#### 3.1.1. Accuracy of Detection (ACCD)

Accuracy of detection (ACCD) is given by Equation (10):

(10) $$\mathrm{ACCD}=\circ \;\circ \;=\frac{T_{N}+T_{P}}{T_{P}+F_{P}+T_{N}+F_{N}}\times 100$$

where $T_{P}$, $F_{N}$, $T_{N}$ and $F_{P}$ represent true positives, false negatives, true negatives, and false positives, respectively.

#### 3.1.2. Logarithmic Loss

The logarithmic loss (*Log Loss*) is utilised with multiple class classifications. It provides an assessment of false classifications in the dataset. Given *N* samples belonging to *M* classes, the log loss is computed using (11) [36].

(11) $$LogLoss=\frac{-1}{N}\sum_{a=1}^{N}\sum_{b=1}^{M}Z_{ab}\times \log(p_{ab})$$

where *Z_ab_* indicates if the sample belongs to class a or b or not and $p_{ab}$ denotes the probability that sample (*a*) belongs to class (*b*). Log loss values closer to zero indicate a higher level of accuracy.

#### 3.1.3. Receiver Operating Characteristic (ROC) Curve

**Figure 8:**
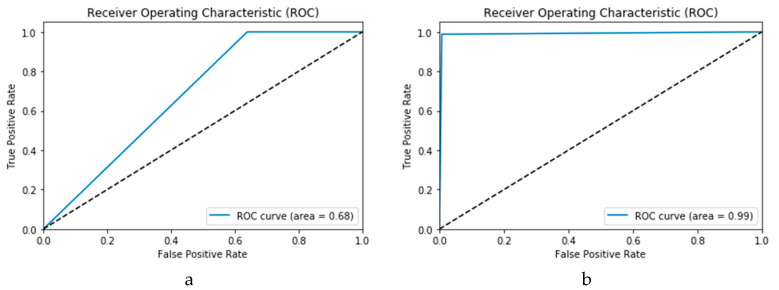
Visualisation of performance indicators for ROC curves. (**a**) Low performance. (**b**) High performance.

The Receiver Operating Characteristic (ROC) curve is utilised as another evaluation metric to provide an accurate visualisation of the simulation results. Within a ROC curve, the $T_{P}$ rate (sensitivity) is represented as a function of the $F_{P}$ rate (specificity) at distinct cut-off points [37]. Every point on the ROC curve illustrates a sensitivity/specificity pair congruent to a specific decision threshold. A test with good discrimination (i.e., no overlap in the two distributions) implies that the ROC curve passes through the upper left corner (i.e., 100% sensitivity, and 100% specificity). Therefore, the nearer the ROC curve to the upper left corner is, the higher the overall accuracy of the test. The accuracy of the proposed technique is indicated by the area under the curve under the ROC curve (AROC). Both the high and low performance are visualised in the ROC curves in Figure 8.

#### 3.1.4. Testing the Execution Time

The Test Execution Time (TET) is a metric to represent the mean time taken to test the input images for (*k*) rounds of the testing operation. 

### 3.2. Simulation Results 

Our experiments are designed to assess the impact of data augmentation on the accuracy of COVID-19 detection. Therefore, execution is carried out via a cohort of two experiments: one with prior data augmentation and the other without it. The former implies direct use of machine learning techniques and deep learning models for the detection. 

In this study, Support Vector Machines (SVMs) and *k*-nearest neighbour (*k*-NN) machine learning techniques are employed as machine learning (ML) techniques. The SVM is implemented with a search grid using a sigmoid gamma function kernel for training, while the *k*-NN is implemented with number of neighbours of five and uniformly weighted functions for training [38]. There are several techniques for *k*-NN, such as KD-tree [39], fast library for approximate nearest neighbours (FLANN) [40], cover tree [41], and semi-convex hull tree [42]. However, this study implements *k*-NN using the brute-force, because of its competitive performance prior to neighbour search for small data samples. This consideration is important for our application since the COVID-19 image datasets are quite small (i.e., without augmentation). Furthermore, our choice is reinforced by the fact that brute-force *k*-NN exhibit high performance as presented in several image processing applications [34,42,43]. 

In the second set of experiments devoid of data augmentation, we assess the performance of CNN and and ConvLSTM DLMs in COVID-19 detection. The DLMs are carried out on a huge training data in order to construct a sufficient feature hierarchy that represents the input data. A small input data cannot satisfy this priority. Therefore, data augmentation is needed to obtain a sufficient amount of the input data in order to construct a satisfactory feature map. Consequently, data augmentation is important for the learnability of the DLMs, which in turn influences optimum classification and performance in COVID-19 detection.

The second cohort of our experiments assesses the impact of prior data augmentation on the performance of the DLMs. Further, as outlined in the previous section, the proposed DLMs are implemented for detection of COVID-19. First, data augmentation is performed on the input data in order to generate a sufficient amount of data. Subsequently, both proposed CNN and ConvLSTM are used to construct feature maps, after which a GAP layer handles the feature map resulting from the feature extraction and converts it into a feature vector that is fed into the classification layer. Finally, classification is carried out using a dense layer with a SoftMax activation function. The main objective is to build such a DLM that constructs a sufficient feature map hierarchy using both CNN and ConvLSTM. Therefore, an optimum DLM based on CNN or ConvLSTM is evaluated by its performance prior to classification of the desired categories.

In their training phase, the proposed DLMs are assessed using *k*-fold cross validation, whereby the learning process is repeated *k* times to attain the diversity required to validate the learning process. To accomplish this task, the data set is randomly divided into *k* groups (or folds) of approximately equal size. The *k* − 1 groups are subsequently used to train the DLMs, whilst the remaining groups are utilised in validating the training. Both the training and validation groups are shifted through *k* rounds.

**Figure 9:**
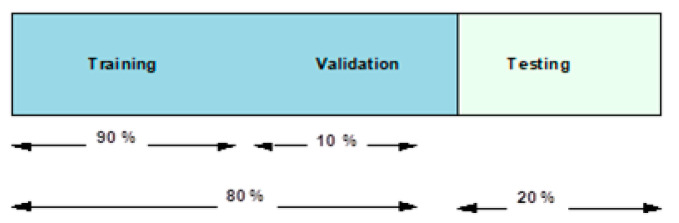
Pipeline for fragmentation of input data.

In our experiments, *k* =10 folds are used, which implies that 90 and 10 percent of the dataset are used in the training and testing over 10 rounds. Furthermore, in each round, ten percent of the testing images are crossed over to the next ten percent. While Figure 9 presents an overview of this data fragmentation pipeline, subsequent subsections present the use of this data fragmentation in the two proposed experimental scenarios as explained earlier.

Throughout the experiments reported, subscripts 1 and 2 will be used to indicate use of the X-ray and CT images, respectively.

#### 3.2.1. COVID-19 Detection Based on Traditional Machine Learning Techniques

**Figure 10:**
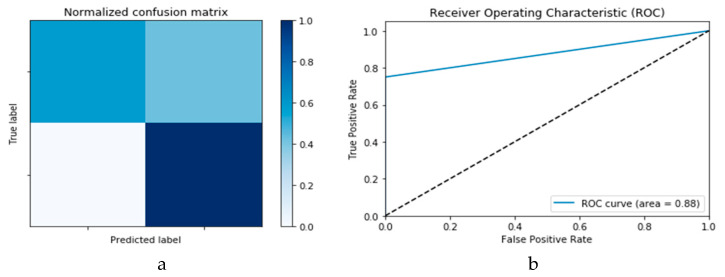
Outcomes for SVM machine learning technique showing (**a**) Confusion matrix and (**b**) ROC curve.

**Figure 11:**
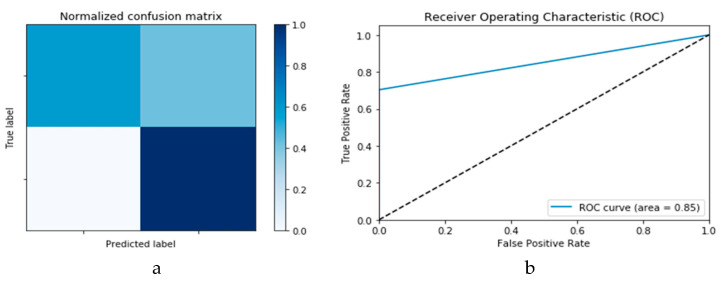
Outcomes for *k*-NN machine learning technique showing (**a**) Confusion matrix and (**b**) ROC curve.

This study also analyses the impact or performance of traditional machine learning techniques for the detection of COVID-19. Both SVM and *k*-NN algorithms are selected based on their high performance in pre-classification tasks and applications in disease diagnosis. Both the SVM and *k*-NN algorithms are executed on the same dataset reported earlier, i.e., X-ray and CT images. Figure 10a,b presents the confusion matrix and ROC curve, respectively, for the SVM technique. As deduced from these curves, accuracy of the SVM technique is 88%. Similarly, Figure 11a,b presents confusion matrix and ROC curve for the *k*-NN algorithm, wherefrom an accuracy of 85% is deduced.

In the remainder of this section, performance evaluation and discussions on outcomes based on the use of DLMs without prior data augmentation are reported as Scenario 1, while Scenario 2 reports the performance based on prior data augmentation followed by the use of the CNN and ConvLSTM DLMs for the two datasets (i.e., X-ray and CT images) used. Finally, we report the performance of the DLMs using CGAN for data augmentation.

#### 3.2.2. Scenario 1: DLMs without Data Augmentation

This experimental scenario typifies the execution of DLMs when challenged with unavailable and/or unreliable data. Its execution provides a basis to assess tenability of the proposed data augmentation process. In other words, Scenario 1 is designed to establish the importance of efficient learning in the performance of DLMs.

**Figure 12:**
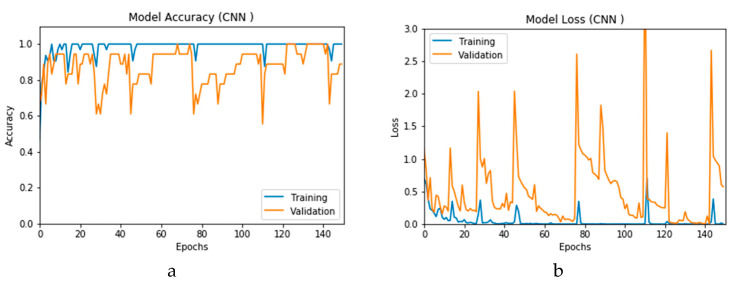
Outcomes for CNN_1_ DLM without data augmentation for the first dataset showing (**a**) Accuracy and (**b**) Logarithmic loss.

**Figure 13:**
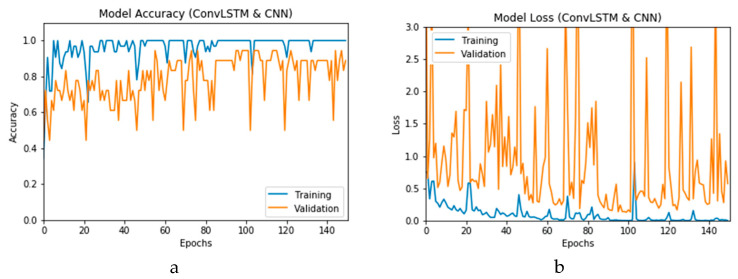
Outcomes for ConvLSTM_1_ DLM without data augmentation for the first dataset showing (**a**) Accuracy and (**b**) Logarithmic loss.

The simulation of this experimental scenario is undertaken using 150 epochs on the input data, which itself is segmented into batches each comprising 10 images. This choice is selected after several trial and error iterations. Figure 12a and b present the outcomes of Scenario 1 for the CNN and ConvLSTM DLMs, respectively. The training and validation curves indicate unstable and precipitated variability. The instability exhibited in these curves illustrates the impact of scarcity (or inadequacy) of input data, which leads to an unstable performance of the DLMs. Additionally, the training and validation curves show an oscillation between 60% and 80% for the proposed CNN-based DLM, while it decreases from 80% to 40% in the ConvLSTM DLM. Furthermore, the loss function for the CNN and ConvLSTM DLMs are presented in Figure 13a and b, respectively. These curves display non-smooth but degrading variations through both the training and validation stages. Moreover, few spikes indicate over-fitting through both the training and validation phases and reveal instability. The precipitated oscillations indicate the impact of the absence of data augmentation in the training process.

**Figure 14:**
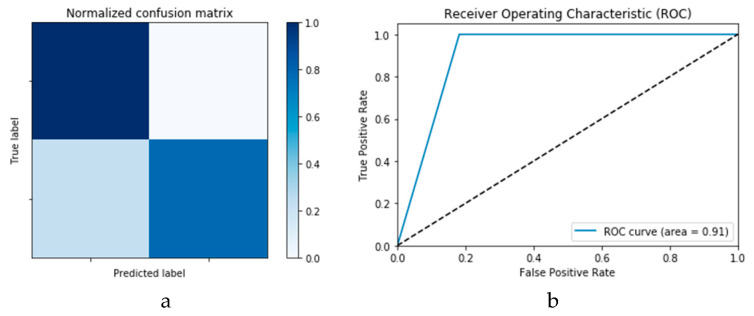
Outcomes for CNN_1_ DLM without data augmentation for the first dataset showing (**a**) Confusion matrix and (**b**) ROC curve.

Figure 14a,b presents the confusion matrices for the True Positive Rate (TPR) and True Negative Rate (TNR), False Positive Rate (FPR) and False Negative Rate (FNR) classification for the CNN and ConLSTM DLMs, respectively. The TPR represents the success of predicting a COVID-19 image truly as a COVID-19 positive image, while FPR represents the failure of this case and of predicting it as a normal image. On the other hand, the TNR represents the success of predicting a normal image as a COVID-19 negative (i.e., Normal), while FNR presents the failure of this and predicting it as a COVID-19 positive image. 

**Figure 15:**
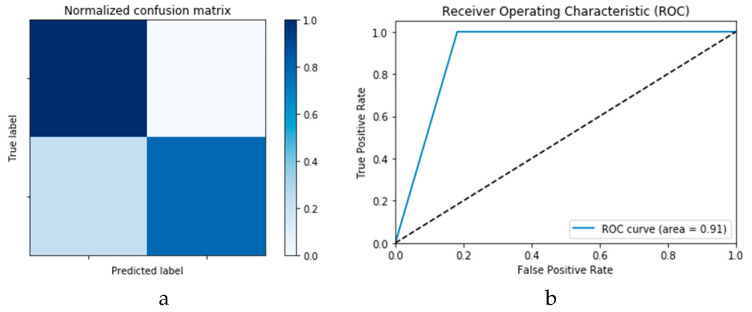
Outcomes for ConvLSTM_1_ DLM without data augmentation for the first dataset showing (**a**) Confusion matrix and (**b**) ROC curve.

Finally, Figure 15a,b presents the ROC curves for the CNN and ConvLSTM DLMs based on experimental Scenario 1. Despite the unstable nature of the training and validation states described earlier, these curves reveal appreciable performance in accuracy as high as 91% for the CNN and ConvLSTM DLMs without prior data augmentation. Nevertheless, this outcome does not meet the thresholds expected for such applications. Moreover, the learning process is predicated on a slender dataset. Table 1 presents the values of accuracy and loss at different iterations for both CNN and ConvLSTM DLMs. 

#### 3.2.3. Scenario 2: DLMs with Prior Data Augmentation Using Image Transformations (DADLM)

In the second experimental scenario, we assess the impact of infusing a prior pixel-based image transformation data argumentation technique to precede execution of both DLMs (i.e., CNN and ConvLSTM). Here, simple image operations including rotation, scaling, blurring, and flipping transforms are utilised for data augmentation. 

**Figure 16:**
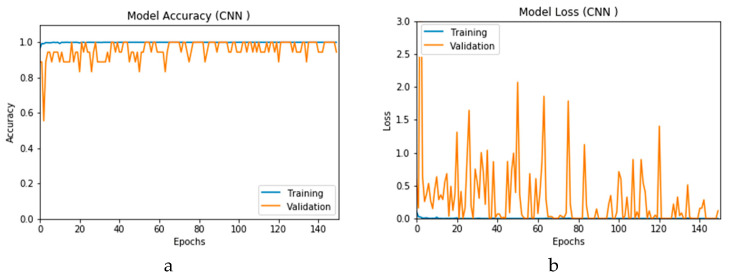
Outcomes for CNN_1_ DADLM with prior data augmentation for the first dataset showing (**a**) Accuracy and (**b**) Logarithmic loss.

Figure 16a,b presents the accuracy for both the CNN- and ConvLSTM-based DLMs with data augmentation. As seen from these outcomes, contrary to Scenario 1, the curves exhibit some stability. Furthermore, we note that the accuracy oscillates in the range of 80% to 99% and 60% to 99% for the CNN- and ConvLSTM-based DLMs, respectively. Additionally, contrary to Scenario 1, little or no variations are visible in the training set curves. Generally, training and validation can be easily tracked, which makes the performance reliable.

**Figure 17:**
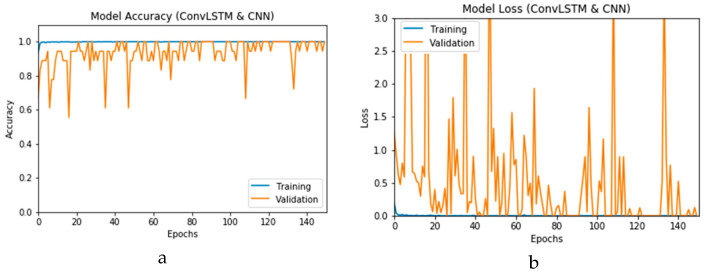
Outcomes for ConvLSTM_1_ DADLM with prior data augmentation for the first dataset showing (**a**) Accuracy and (**b**) Logarithmic loss.

Figure 17a,b presents the loss curves for the two models with data augmentation. Much like the accuracy analysis, we see that, unlike Scenario 1, despite the degradations during training epochs, the curves exhibit less variations. The loss curve for the CNN-based DLM is degraded from 3 to 0.25, while that of the ConvLSTM-based DLM appears relatively complicated, because the original data contains a subset of the images used for training. This is an indicator that, although the data augmentation is performed, the provided features from the data are not sufficient for superior training. 

**Figure 18:**
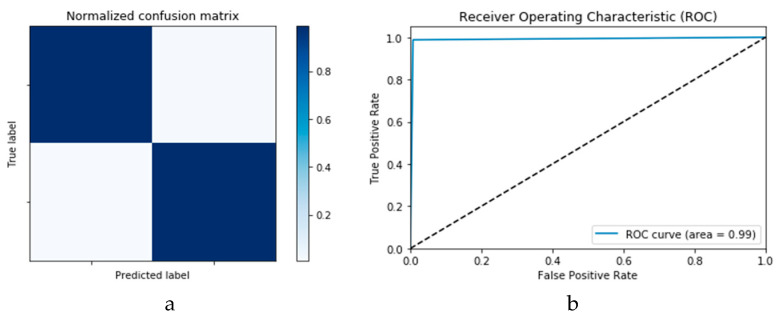
Outcomes for CNN_1_ DADLM with prior data augmentation for the first dataset showing (**a**) Confusion matrix and (**b**) ROC curve.

**Figure 19:**
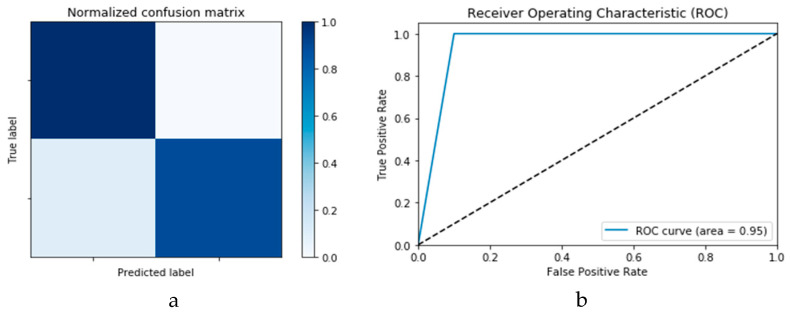
Outcomes for ConvLSTM_1_ DADLM with prior data augmentation for the first dataset showing (**a**) Confusion matrix and (**b**) ROC curve.

Figure 18a,b presents the confusion matrices for the two models based on the second experimental scenario. Similarly, Figure 19a,b presents the ROC curves for the two DLMs. Therefore, we deduce accuracies of 99% and 95% for the CNN-based and ConvLSTM-based DLMs, respectively. Compared with the accuracy obtained in Scenario 1, both models show better efficiency. Furthermore, the outcome validates earlier claims regarding the impact of prior data augmentation as the trio of accuracy, logarithmic loss, and ROC metrics show marked improvements relative to those reported in Scenario 1.

#### 3.2.4. Analysis of the Impact of the Proposed DADLM on the Second Dataset

**Figure 20:**
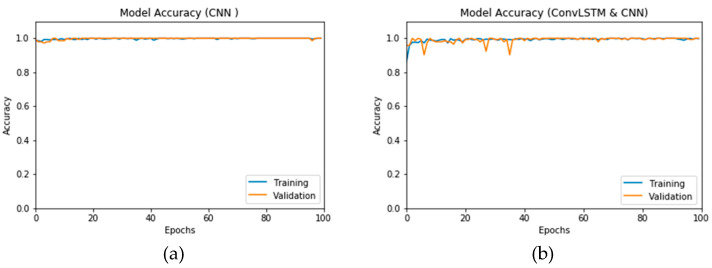
Accuracy for DADLMs with prior data augmentation for the second dataset. (**a**) CNN_2_ DADLM and (**b**) ConvLSTM_2_ DADLM.

Figure 20a,b presents the accuracy of the proposed DLMs on a dataset demarcated into two categories, each of comprising of 288 images for COVID-19 positive (i.e., infected) and negative (i.e., normal) classifications. Much like previous implementations of the DADLM data augmentation, the image transformations described in Section 2 are used to proliferate these sub-datasets by 10 folds, i.e., to 2880 images in each category. However, in contrast to the outcomes reported for the first dataset, the curves presented (i.e., Figure 20) are quite stable. The accuracy curve oscillates in the range from 80% to 99% for the CNN-based DADLM (i.e., CNN_2_ DADLM). On the other hand, it oscillates between 60% and 99% for the validation, i.e., testing, curves for the ConvLSTM DADLM (i.e., ConvLSTM_2_ DADLM). Similarly, marginal variations are observed for the training set. Therefore, it can be surmised that the tracking of the training and validation is reliable since stability is observed in both curves.

**Figure 21:**
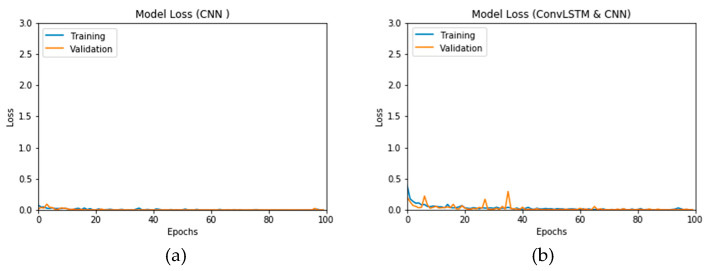
Logarithmic loss for DADLMs with prior data augmentation for the second dataset. (**a**) CNN_2_ DADLM and (**b**) ConvLSTM_2_ DADLM.

Figure 21a,b presents the logarithmic loss curves for the second dataset. Therefrom, we can deduce that the curves exhibit less variations than those of the previous dataset with the logarithmic loss values degrading during the training epochs. The logarithmic loss curve for the CNN_2_ DADLM degrades from 0.5 to zero, while that for the ConvLSTM_2_ DADLM (i.e., in Figure 21b) presents a more complicated outcome. 

**Figure 22:**
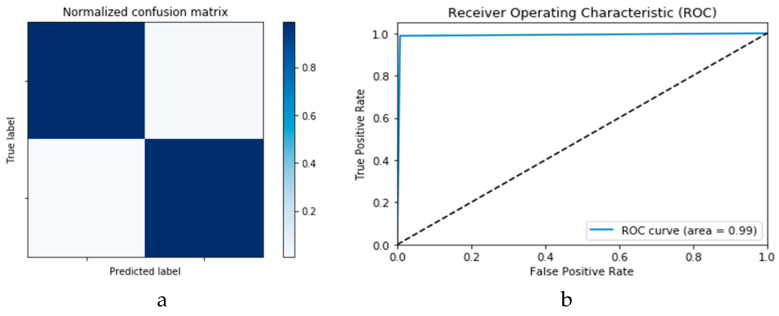
Outcomes for CNN_2_ DADLM with prior data augmentation for the second dataset showing (**a**) Confusion matrix and (**b**) ROC curve.

**Figure 23:**
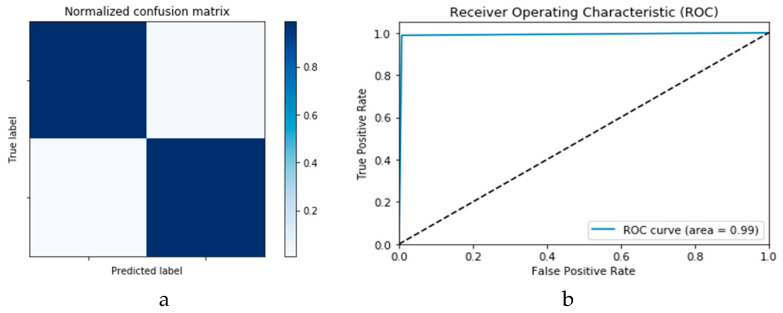
Outcomes for ConvLSTM_2_ DADLM with prior data augmentation for the second dataset showing (**a**) Confusion matrix and (**b**) ROC curve.

**Figure 24:**
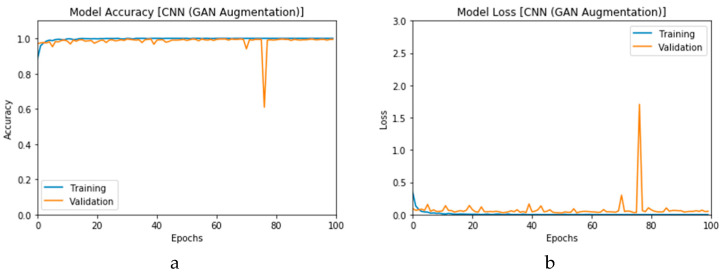
Outcomes for CNN_1_ CGAN DADLM with prior GAN data augmentation for the first dataset showing (**a**) Accuracy and (**b**) Logarithmic loss.

**Figure 25:**
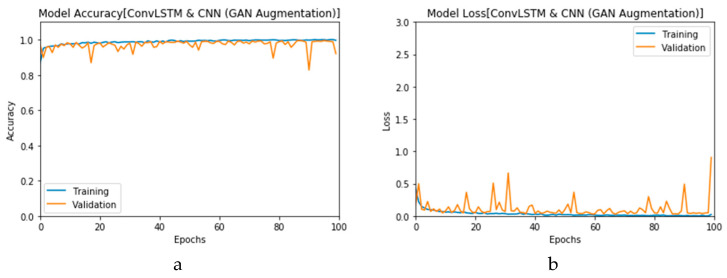
Outcomes for ConvLSTM_1_ CGAN DADLM with prior GAN data augmentation for the first dataset showing (**a**) Accuracy and (**b**) Logarithmic loss.

The confusion matrices and ROC curves reported in Figure 22 and Figure 23 present the performance of the proposed CNN and ConvLSTM DADLMs based on the second dataset, i.e., CNN_2_ and ConLSTM_2_. Specifically, from Figure 23a,b, we see that both DADLMs have accuracy reaching 99%. These outcomes are better than those reported for Scenarios 1 and 2 using the previous dataset. Furthermore, unlike the results reported for the first dataset, here, the curves support the deduction that both the training and validation tests are more stable and reliable. The stability is traced to the distended dataset size arising from our proposed data augmentation. These findings validate the earlier claims regarding the impact of data augmentation on enhancing the accuracy of image-based detection of COVID-19. Additionally, Figure 24 and Figure 25 present the confusion matrices and ROC curves for the CNN_2_ and ConvLSTM_2_ DADLMs, respectively. Like the previous results, the baseline of 99% accuracy is reported throughout. 

#### 3.2.5. DLMs with Prior Data Augmentation Based on CGAN (CGAN DADLM)

This section presents an analysis of the performance of the proposed DLMs with data augmentation using the convolutional generative adversarial network (CGAN) introduced earlier in Section 2. The proposed DLMs with CGAN data augmentation are implemented on both datasets, i.e., the X-ray and CT images. Like its previous use, throughout the experiments reported, subscripts 1 and 2 will be used to denote use of the X-ray and CT images, respectively.

**Figure 26:**
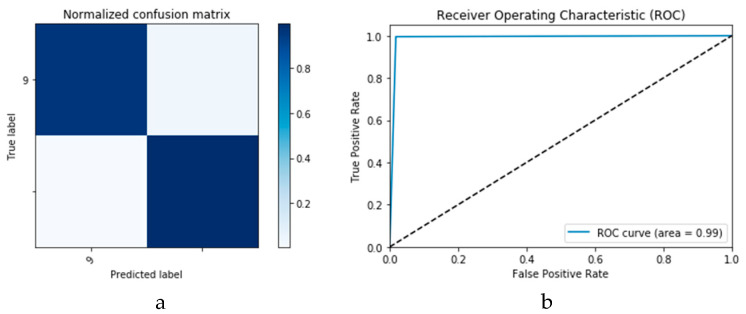
Outcomes for CNN_1_ CGAN DADLM with prior data augmentation for the first dataset showing (**a**) Confusion matrix and (**b**) ROC curve.

**Figure 27:**
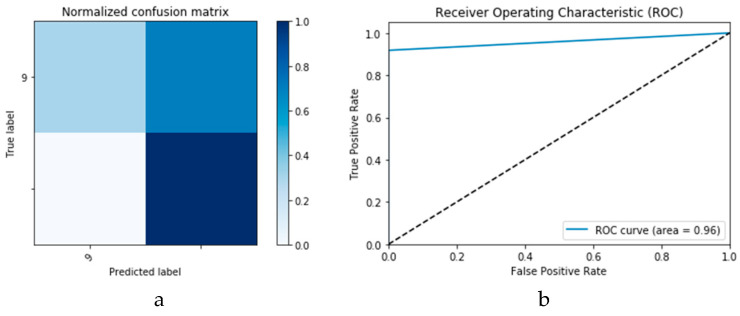
Outcomes for ConvLSTM_1_ CGAN DADLM with prior data augmentation for the first dataset showing (**a**) Confusion matrix and (**b**) ROC curve.

First, the proposed modalities are performed on X-ray images whose results for training and validation phases along the epochs (100 epoch) of the proposed CNN model with GAN data augmentation (i.e., CNN_1_ CGAN DADLM) are presented in the curves in Figure 24a and b, respectively. Similarly, Figure 25a and b present the curves for accuracy and logarithmic loss for both training and validation phases of the proposed ConvLSTM with GAN data augmentation (i.e., ConvLSTM_1_ CGAN DADLM). In addition, Figure 26 presents the confusion matrix and ROC curve for the proposed CNN_1_ CGAN DADLM, while Figure 27 presents similar confusion matrix and ROC curve for the proposed ConvLSTM_1_ CGAN DADLM. From these results, we see that the proposed CNN_1_ CGAN DLM records a testing accuracy of 99%, while the proposed ConvLSTM_1_ CGAN DADLM achieved an accuracy of 96%.

**Figure 28:**
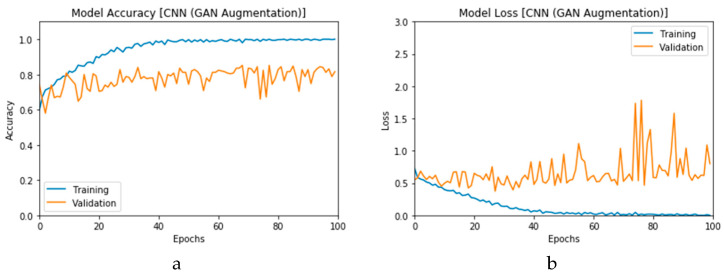
Outcomes for CNN_1_ CGAN DADLM with prior GAN data augmentation for the second dataset showing (**a**) Accuracy and (**b**) Logarithmic loss.

**Figure 29:**
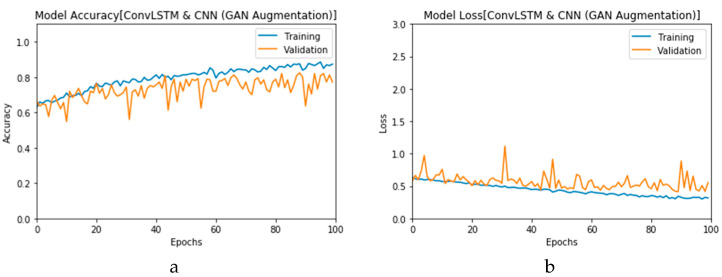
Outcomes for ConvLSTM_1_ CGAN DADLM with prior CGAN data augmentation for the second dataset showing (**a**) Accuracy (**b**) Logarithmic loss.

**Figure 30:**
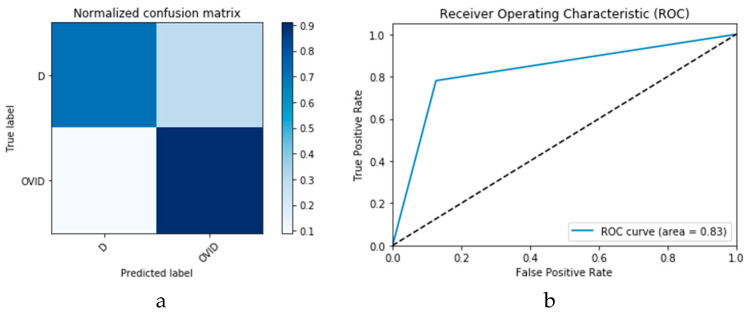
Outcomes for CNN_1_ CGAN DADLM with prior CGAN data augmentation for the second dataset showing (**a**) Confusion matrix and (**b**) ROC curve.

**Figure 31:**
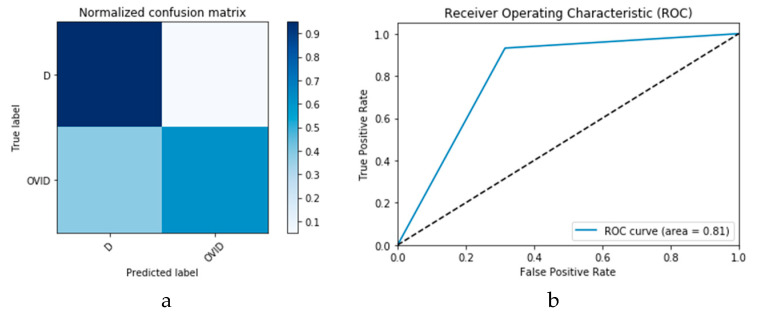
Outcomes for ConvLSTM_1_ CGAN DADLM with prior CGAN data augmentation for the second dataset showing (a) Confusion matrix and (**b**) ROC curve.

Furthermore, the proposed CGAN DADLMs are implemented on our second datasets, i.e., the CT images. Figure 28a,b presents curves for training and validation phases of the proposed CNN_2_ CGAN DADLM, while Figure 29a,b presents similar curves for accuracy and logarithmic loss for both training and validation phases for the proposed ConvLSTM_2_ CGAN DADLM. Additionally, Figure 30 presents confusion matrix and ROC curve for the proposed CNN_2_ CGAN DADLM, while Figure 31 presents the confusion matrix and ROC curve for the proposed ConvLSTM_2_ CGAN DADLM. From these results, we deduce that the proposed CNN_2_ CGAN DADLM records a testing accuracy of 83%, while an accuracy of 81% is reported for the proposed ConvLSTM_2_ CGAN DADLM.

## 4. Discussion

Given a limited dataset, the experiments reported in the preceding section sought to assess the impact of data augmentation on image-based detection of COVID-19 as an important step to assist doctors and other specialists in making conclusive diagnosis regarding COVID-19 status of patients. 

Based on this objective, the experiments involved performance analysis along two broad boundaries, i.e., presence and absence of data augmentation. Furthermore, this analysis was predicated on the type of learning strategy employed, i.e., whether machine or deep learning. In addition, two algorithmic frameworks were used to perpetuate both learning strategies, i.e., support vector machine (SVM) and *k*-nearest neighbour (*k*-NN) for the machine learning (ML) techniques and convolutional neural networks (CNN) and convolutional long short-term memory (ConvLSTM) for the deep learning models (DLMs). Further, these learning strategies are implemented on two datasets comprising X-ray and computed tomography (CT) images. 

The flow of the experiments reported was to transition from analysis without data augmentation starting with ML techniques and subsequently to implementation of DLMs with prior data augmentation. Data augmentation is accomplished via two strategies. First, a simple set of image transformations were used to distend the size of the two datasets. Second, the more advanced convolutional generative adversarial network (CGAN) was used to proliferate the two datasets. 

**Figure 32:**
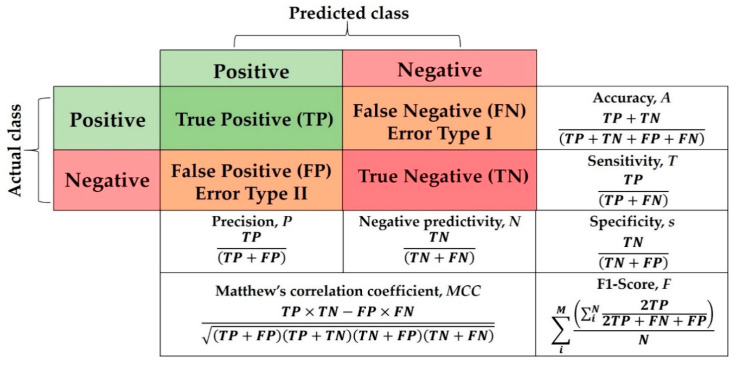
Equation matrix for binary quality metrics.

For a fair and objective performance assessment, the CGAN data augmentation binary metrics specified in [29], which include sensitivity (s), specificity (T), positive predictive value (i.e., PPV or True Positive (TP)), negative predictive value (i.e., NPV or True Negative (TN)), Accuracy (A), F1-score (F), which is the harmonic mean of precision and sensitivity, and Matthews correlation coefficient (MCC)), are used to evaluate our proposed DADLMs. These metrics are presented in the binary quality metrics equation matrix in Figure 32. As deduced from that figure, both MCC and Accuracy (A) take into consideration the TP, FP, TN and FN, whereas MCC is generally regarded as a balanced measure that can be used even if classes are of different sizes [44], Accuracy takes into account the number of sets applied in the five-fold and the number of classes, i.e., *M* and *N*.

Table 2 presents a summary of outcomes of six out of the seven binary quality metrics for our models (i.e., excluding the detection accuracy, which is reported and discussed separately). From these results, we see (best four entries for each metric highlighted in bold) that the two proposed data-augmented models, i.e., DADLM and CGAN DADLM outperform all the other methods. Further, between them, the image-based transformations data-augmented DLM, i.e., DADLM, performs better with the CT images while the CGAN data-augmented DLM, i.e., CGAN DADLM, presents better performance with the X-ray images used in the experiments reported. In a head to head comparison for the X-ray images in the first dataset, we see that although both CNN_1_ DADLM and CNN_1_ CGAN DADLM presented similar results across all six metrics, the CNN_1_ DADLM has a slight edge. Similarly, for the CT images in the second dataset, head to head evaluation shows that the CNN_2_ DADLM presents the best performance in terms of specificity, PPV, and MCC. Hence, for both datasets, the image-based transformation focused data augmentation DLM edges out all the other models. Metrics such as those presented here provide the conclusions regarding image-based contributions to support doctors and other specialists in making efficient diagnosis of COVID-19.

**Figure 33:**
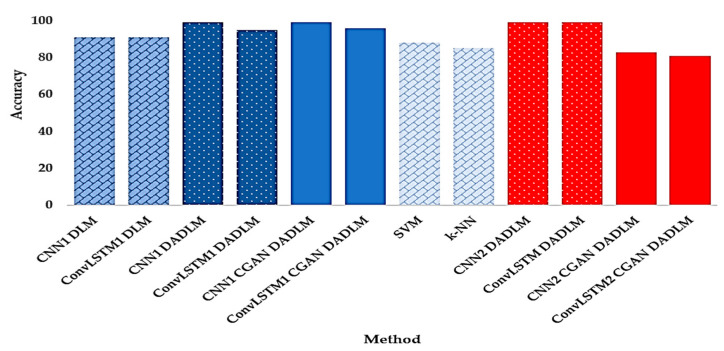
Performance comparison of the proposed DLMs for different datasets.

Additionally, in delivering with the main of the study and to further establish the performance of our data augmentation DLMs, we present, in Figure 33, a summary of the outcomes in terms of detection accuracy for the two datasets, two experimental scenarios and two machine learning techniques as explained above. This chart presents the lucid validation of the impact of data augmentation for the methods reported, where subscripts 1 and 2 indicate the use of the first and second datasets, respectively. To enhance readability of the chart, blocks in different shades of blue denote outcomes based on the first dataset (i.e., X-ray images), while different shades of red indicate results emanating from CT images, i.e., the second dataset. Furthermore, dotted blocks denote our proposed DADLM, i.e., DLM using image transformations for data augmentation, while solid blocks indicate the proposed DLM using CGAN for data augmentation. Finally, diamond brick blocks indicate ML techniques and DLMs without prior data augmentation.

The results reported indicate that, relative to the different DLMs reported, a low performance for the ML techniques with increase in detection accuracy ranging from 11 to 14%. Similarly, focusing on the X-ray images dataset, we note that despite their better performance than the ML techniques, DLMs without data augmentation (i.e., CNN_1_ DLM and ConvLSTM_1_ DLM) fall short of the accuracy reported the image transformations-based versions of both DLMs, i.e., CNN_1_ DADLM and ConvLSTM_1_ DADLM, as well as their CGAN data-augmented versions, i.e., CNN_1_ CGAN DADLM and ConvLSTM_1_ CGAN DADLM.

Remarkably, for the X-ray images dataset, both data-augmented CNN DLMs (i.e., CNN_1_ DADLM and CNN_1_ CGAN DADLM) report best performance with 99% detection accuracy for COVID-19 positive (i.e., infected) and negative (i.e., normal) classes.

While a similar trend is observed in the CT images dataset, the below-par performance of the CGAN data-augmented DLMs (i.e., CNN_2_ CGAN DADLM and ConvLSTM_2_ CGAN DADLM) is noteworthy. The difference of 16 and 18% between the CGAN and image transformations-based data augmentation DADLMs is remarkable. A plausible explanation would be, as with most DLMs, there are losses associated at each layer of the CGAN used in our CGAN DADLM. This causes distortions to subsequent layers which accumulate recursively. In comparison, the pixel-wise, image-based transformations used in our image transformation-based data-augmented DLM, i.e., DADLM, is immune to these types of losses.

Notwithstanding its remarkable performance in the CT images dataset, incidences of negative false predictions were observed with the image transformation-based data-augmented DLM (i.e., DADLM). These misclassifications are attributed to loss of image details during transformations used, especially from the impact of low resolution on the rescaling operation. Interestingly, there are more occurrences of these misclassifications in results from the X-ray images than from the CT images dataset.

Finally, to extrapolate the impact of the data augmentation strategies proposed in this study, we present, in Table 3, a comparison with similar efforts. Constrained by limited number of such studies and considering the incidence of pneumonia in COVID-19 diagnosis, we include studies focussed on image-based detection of pneumonia in our performance analysis. Motivation for this stems from a report from the WHO that earliest cases of COVID-19 were reported as pneumonia and, even today, the most common diagnosis of severe COVID-19 is severe pneumonia. Therefore, we surmise, this should not significantly impact any conclusions made.

Moreover, we also include comparisons with [29,48] that report COVID-19 detection based on X-ray images. We further note that [48,49] make use of GAN in data augmentation, which makes them ideal like for like platforms to evaluate the performance of our proposed DADLMs. To provide a level playing ground, we limit the comparison to the X-ray images dataset. Therefore, we report only results from CNN_1_ DADLM, ConvLSTM_1_ DADLM, CNN_1_ CGAN DADLM, and ConvLSTM_1_ CGAN DADLM.

As observed from Table 3, notwithstanding differences in deployment, our proposed data augmentation strategies, i.e., DADLM and CGAN DADLM, match or outperform the reported competitors. In fact, with 99% average accuracy in detection of COVID-19 positive (i.e., infected) and negative (i.e., normal) cases, the proposed data augmentation strategies using CNN DLM (i.e., CNN_1_ DADLM and CNN_1_ CGAN DADLM) outperform most of the methods reported.

## 5. Concluding Remarks

Faced with an existential crisis, mankind must pull together solidarity and technology to save itself. While medical and healthcare providers are the first line of defence against the Corona virus (COVID-19) pandemic, engineers, scientists, and other professionals all have a role to play in defeating this common enemy. Over time, machine and deep learning tools have emerged as veritable tools to enhance and improve technologies across all domains and applications, including disease detection, diagnosis, treatment, and cure. The study presented explored deployment of these tools in supporting the battle against COVID-19. While this would seem instinctive, another challenge arises because of inadequate access to image-based data from patients during and after the infection, i.e., recoveries. To overcome both challenges, our study presented a data augmentation framework that distends the limited dataset of X-ray and CT images by 100%. This enhanced dataset was subsequently used to improve the learnability of the proposed deep learning models (DLMs). We analysed performance of our strategy alongside two scenarios with and without data augmentation for two datasets of different sizes. Furthermore, we compared the performance of the DLMs against traditional machine learning techniques employing the SVM and *k*-NN algorithms. Across all metrics reported, our proposed data-augmented DLMs outperformed the other approaches. In terms of detection accuracy for COVID-19, the proposed DADLM technique presented improvements between 4% and 8% compared to similar DLMs devoid of data augmentation. This increases to the range from 11% to 14%, when compared to the SVM and *k*-NN machine learning techniques. In a head to head comparison between our two proposed data augmentation strategies, the image-based transformations DLM (i.e., DADLM) edges out the CGAN data-augmented DLM (CGAN DADLM) in terms of seven binary classification quality metrics (i.e., sensitivity specificity, positive predictive value (i.e., True Positive (TP)), negative predictive value (i.e., True Negative (TN)), Accuracy, F1-score, and Matthews correlation coefficient) for both datasets used in the experiments reported. However, compared to recent studies reporting image-based detection of COVID-19 and pneumonia, both our DADLMs, i.e., the pixel-wise image-based transformations and CGAN data augmentation strategies, show superior performance. Metrics such as those presented in the study provide useful conclusions regarding image-based contributions to support doctors’ diagnosis of COVID-19.

In the ongoing work, additional image-based modalities are being sourced to expand the width (i.e., in terms of availability) as the depth (in terms of reliability) expands. The proposed models will be refined to further enhance accuracy. Finally, other health informatics will be integrated to develop a more robust DLM framework for efficient COVID-19 management covering containment, mitigation, identification, tracking as well as disease detection, diagnosis, and treatment. With our humanity and ability to exploit our technological advances, we will collectively defeat this scourge and use today’s experiences to prepare for future similar battles when (not if) they come.

## Figures and Tables

**Table 1:** Accuracy and logarithmic loss for different iterations for CNN and ConvLSTM DMLs.

Epochs	CNN		ConvLSTM	
	Accuracy	Loss	Accuracy	Loss
10	72	2.623	65.3	1.9612
20	79	1.664	67.8	2.463
30	79.33	1.5187	79.3	1.159
40	73.4	2.919	73.5	2.205
50	0.553	87.5	87.5	0.553
60	74.4	3.035	75.2	1.396
70	87.2	0.9277	78.5	0.997
80	69.7	3.559	72	2.807
90	72.3	2.779	72.3	2.479
100	82.5	1.639	82.5	1.639
110	73.9	3.366	72.8	3.126
120	76.5	2.563	76.5	2.563
130	77.5	2.289	87.5	1.047
140	81	1.958	89	0.992
150	91	0.559	91	0.541

**Table 2:** Outcomes of binary classification quality metrics for proposed DADLMs and traditional ML techniques (all results expressed as percentages).

Model	Sensitivity	Specificity	PPV	NPV	F1-Score	MCC
CNN_1_ DLM	98.7	83.6	85.7	98.5	91.7	83.2
ConvLSTM_1_ DLM	98.4	83.3	85.7	98.0	91.6	82.7
CNN1 DADLM	**99.7**	**98.7**	**98.7**	**99.7**	**99.0**	**98.4**
ConvLSTM_1_ DADLM	**100**	90.1	91.0	**100**	95.3	90.6
CNN_2_ DADLM	99.7	**98.7**	**98.7**	99.7	**99.0**	**98.4**
ConvLSTM_2_ DADLM	99.6	**98.6**	**98.6**	99.6	**99.0**	98.1
SVM	95.5	80.7	83.1	94.7	88.8	76.9
*k*-NN	95.5	74.3	79.5	94.0	86.7	71.6
CNN_1_ CGAN DADLM	**100**	**97.8**	**97.7**	**100**	**99.0**	**97.7**
ConvLSTM_1_ CGAN DADLM	**100**	92.4	91.6	**100**	95.7	92.0
CNN_2_ CGAN DADLM	87.5	80.0	75.0	90.0	80.7	66.4
ConvLSTM_2_ CGAN DADLM	87.1	74.1	79.4	83.3	83.1	61.9

**Table 3:** Comparison of average detection accuracy from proposed models alongside those from traditional and recent methods.

Model	Year	Implementation	Description	Accuracy (%)
Proposed	2020	X-ray and CT images for COVID-19 detection	CNN_1_ DADLM	**99.0**
			ConvLSTM_1_ DADLM	95.0
			CNN_1_ CGAN DADLM	**99.0**
			ConvLSTM_1_ CGAN DADLM	**96.0**
[45]	2018	X-ray images (pneumonia)	CNN	92.8
[46]	2019	X-ray images (pneumonia)	CNN + 2 Dense Layers + Augmentation	93.7
[19]	2019	X-ray images (pneumonia)	CNN + 3 Dense Layers	95.3
[47]	2019	X-ray images (pneumonia)	CNN + 2 Dense Layers	96.7
[23]	2020	X-ray images (pneumonia)	CNN + Random Forest	97.0
[29]	2020	X-ray images (COVID-19)	CNN + Actual data + Synthetic Augmentation	95.0
[48]	2020	X-ray images (COVID-19)	Alexnet + GAN data augmentation	80.6
			Googlenet + GAN data augmentation	85.2
			Resnet18 + GAN data augmentation	**100**
[49]	2020	X-ray images (pneumonia)	Alexnet + GAN data augmentation	96.1
			Squeeznet + GAN data augmentation	97.8
			Google + GAN data augmentation	96.8
			Resnet18 + GAN data augmentation	99.0
